# AMPDeep: hemolytic activity prediction of antimicrobial peptides using transfer learning

**DOI:** 10.1186/s12859-022-04952-z

**Published:** 2022-09-26

**Authors:** Milad Salem, Arash Keshavarzi Arshadi, Jiann Shiun Yuan

**Affiliations:** 1 Electrical and Computer Engineering Department, University of Central Florida, Orlando, FL USA; 2 Burnett School of Biomedical Sciences, University of Central Florida, Orlando, FL USA

**Keywords:** Antimicrobial peptide, Deep learning, Drug discovery, Hemolysis, Transformers

## Abstract

**Background:**

Deep learning’s automatic feature extraction has proven to give superior performance in many sequence classification tasks. However, deep learning models generally require a massive amount of data to train, which in the case of Hemolytic Activity Prediction of Antimicrobial Peptides creates a challenge due to the small amount of available data.

**Results:**

Three different datasets for hemolysis activity prediction of therapeutic and antimicrobial peptides are gathered and the AMPDeep pipeline is implemented for each. The result demonstrate that AMPDeep outperforms the previous works on all three datasets, including works that use physicochemical features to represent the peptides or those who solely rely on the sequence and use deep learning to learn representation for the peptides. Moreover, a combined dataset is introduced for hemolytic activity prediction to address the problem of sequence similarity in this domain. AMPDeep fine-tunes a large transformer based model on a small amount of peptides and successfully leverages the patterns learned from other protein and peptide databases to assist hemolysis activity prediction modeling.

**Conclusions:**

In this work transfer learning is leveraged to overcome the challenge of small data and a deep learning based model is successfully adopted for hemolysis activity classification of antimicrobial peptides. This model is first initialized as a protein language model which is pre-trained on masked amino acid prediction on many unlabeled protein sequences in a self-supervised manner. Having done so, the model is fine-tuned on an aggregated dataset of labeled peptides in a supervised manner to predict secretion. Through transfer learning, hyper-parameter optimization and selective fine-tuning, AMPDeep is able to achieve state-of-the-art performance on three hemolysis datasets using only the sequence of the peptides. This work assists the adoption of large sequence-based models for peptide classification and modeling tasks in a practical manner.

## Background

Historically, natural products have been of the most important candidate drug sources in the pharmaceutical industry. Due to millions of years of evolution and constant updates, they have been optimized in defending the host microorganism against pathogens. One source of natural products is the innate immune system of animals, plants, and fungi [1], where peptides have been discovered to have antimicrobial and antibacterial properties. These natural products have inspired the discovery and design of Antimicrobial peptides (AMPs), a group of short peptides known for their potency against viruses, bacteria, fungi, and transformed cancer cells. High specificity and selectivity, low toxicity, and high diversity are their privileges over other classes of drugs in spite of their short lifetime and low bio-availability [2, 3]. These peptides largely work as immunomodulators, apoptosis stimulators, and proliferation inhibitors [1]. Disrupting the structure of cells or mitochondria membrane and inhibiting DNA or Protein synthesis and their interactions are mechanisms that have been observed for the effectiveness of AMPs [2, 3]. There are several examples of these peptides including Magainin, Pleurocidin, Buforins as membrane disturbers and BR2, NGR, Tat-KLA, K6L9, and HNP as intracellular effectors [2, 3]. One advantage of AMPs is their potency among different types of cancers based on their membrane features [3].

However, one main challenge that hinders the adoption of AMPs as therapeutics is that AMPs are prone to be hemolytic. Hemolysis happens when the peptide ruptures red blood cells, ending the life-time of these cells prematurely. This is an unintended side-effect and due to its severity, a hemolytic peptide cannot be an applicable drug candidate. As an estimate, it is predicted that 70% of all known AMPs have a high or moderate hemolytic activity [4]. Therefore, investigation of hemolysis and prediction of hemolytic activity in therapeutic peptides is necessary for the development of AMPs as hit candidates.

Fortunately in recent years, multiple efforts have been made to collect databases of AMPs, as well as to classify their hemolytic activity through computational methods. The dominant approach for classification of AMPs includes extracting physicochemical features from the peptide, effectively turning peptides into a feature vector, and classifying them with a machine learning model [5]. The features used and discovered in these classification systems include composition, physicochemical properties and structural characteristics of amino acids, length of the peptide, net charge, hydrophobic percentage, tertiary structure, atom composition, diatom composition, chemical descriptors, fingerprints, and binary profiles [4–8]. The methodology of extracting features from the input and then classifying the feature vectors is akin to the traditional Machine Learning approach in many domains such as Electrocardiogram arrhythmia detection [9], image classification [10], and even antimicrobial peptide identification [11]. In all of these domains, deep learning has shown supremacy in its modeling power via learning features during training from the raw data. This combination of feature extraction and modeling is enabled via deep learning’s automatic feature extraction capability, where useful features are learned for the current training dataset. Deep Learning modeling can identify unknown features in data and discover abstract and hidden patterns within the input data that are important for the classification task at hand.

To this end, there are two works in the literature which aim to tackle the problem of hemolysis activity prediction using deep learning-based models, both of which fail to outperform their feature-based counterparts. First a Recurrent Neural Network (RNN) model has been used to learn from raw peptide sequences alone [12] as part of a peptide generation pipeline, and then tasked to filter the generated peptides that are predicted to be hemolytic. Moreover, ELMO [13], which is a deep learning-based bidirectional language model, has been used in another related work [14] to predict cytotoxicity and hemolysis activity of peptides given a sequence. However, both of these sequence-based and deep learning-based methods were not able to outperform the traditional feature-based approaches in certain benchmarks [12, 14]. The main reason contributing to this shortcoming in performance is the small amount of available training data [14], which is intuitive due to the data-hungry nature of the deep learning-based models and the high cost of automatic feature extraction.

Given the main challenge of small data, in this work we propose the use of transfer learning to alleviate this problem. Transfer learning allows the model to be trained at a source task, then transferred to a target task in the form of initialization which holds the patterns learned from the source task. With this technique, the cost of automatic feature extraction is partially paid in the source domains and the challenge of low data within the target domain is reduced. To this end, AMPDeep thoroughly investigates the effects of initializing a transformer-based model from a pre-trained protein language model, i.e. Prot-BERT-BFD [15], which has been trained on masked amino acid prediction in a self-supervised manner. Having done so, AMPDeep also studies “secretion” as another source for transferring knowledge from, since secretion is a common and biologically important feature of antimicrobial peptides and hemolytic peptides, and a large number of secretory peptides are available as training data. Therefore, transfer learning is leveraged to fine-tune the model on secretory peptide classification, to make the training of the model on the hemolytic prediction tasks more stable. The training pipeline is tested using three different datasets taken as is from external works, as well as a combined datasets compiled from combining the three datasets and cleaning redundant sequences. Due to the large size of the model, selective fine-tuning is introduced, which lowers the number of parameters available for training. The main contributions of this work are as follows:

 Use of a large transformer-based model to learn from peptides in a sequence-wise manner.

- Use of a pre-trained language model trained on 2 billion protein fragments as initialization.
- Use of transfer learning to transfer learned knowledge from secretion classification task to hemolytic activity classification.
- Introduction of a novel algorithm, i.e. selective fine-tuning, where in 8 scenarios certain parameters of the model are kept frozen during fine-tuning, to facilitate training of a large model on a small amount of data.
- Addressing the problem of redundant sequences in related works via compiling a new hemolytic activity-based dataset.
- Outperforming the state-of-the-art deep learning and feature-based models in hemolytic activity classification

## Results

### AMPDeep pipeline overview

**Fig. 1:**
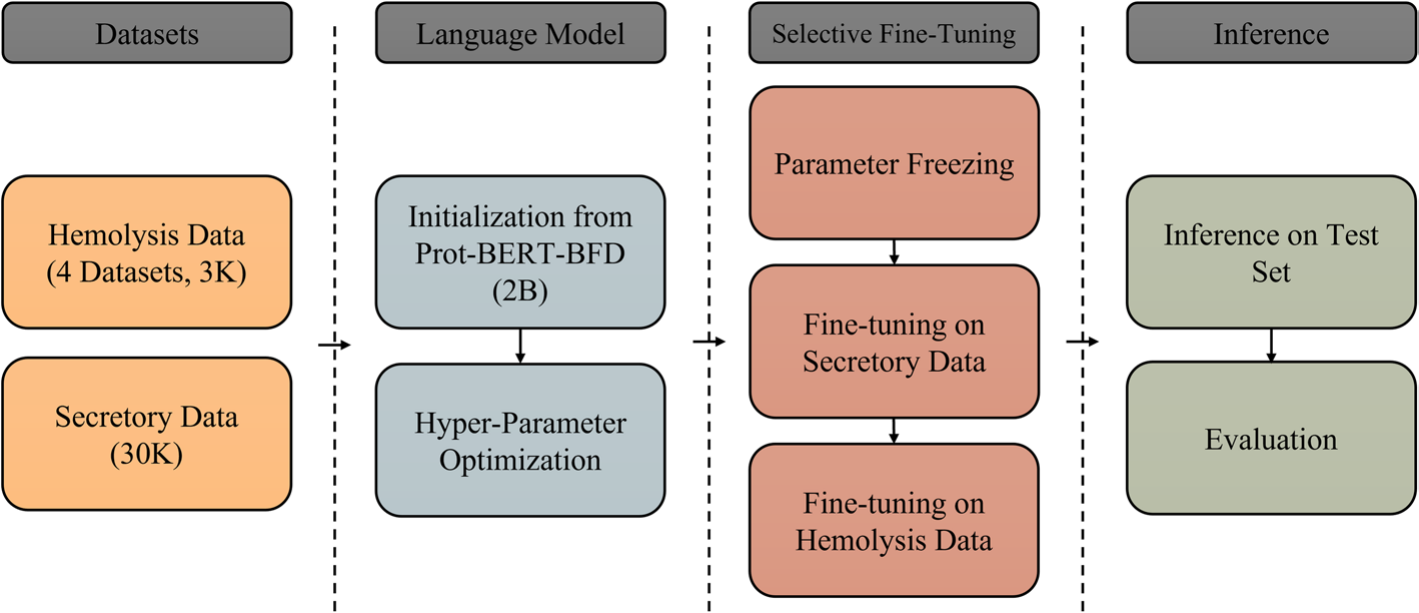
Overview of AMPDeep’s Classification System. Data is gathered for two tasks, i.e. hemolysis and secretion. A pre-trained protein language model is first fine-tuned on secretion detection, then fine-tuned to predict hemolysis activity of a given peptide. The model is evaluated on independent test sets

AMPDeep offers a practical pipeline for hemolytic activity classification of AMPs through transfer learning. To do so, the data is first gathered and cleaned for hemolytic activity classification as well as secretory peptide classification. Next, a massive pre-trained model (Prot-BERT-BFD) [15] is used as initialization for the training. Having done so, hyper-parameter optimization and freezing is performed, which makes the model ready for classification stages. During classification, first, the model is trained on predicting whether a peptide is secretory or not. Then this fine-tuned model is transferred and used as initialization for training whether a given peptide is hemolytic or not. After the model is trained, three external datasets are used to evaluate the performance of the model and enable comparison to related works. This training process is implemented for three hemolysis datasets and a combined dataset. The overview of this work is shown in Fig. 1.

### Data summary

The five datasets used in this work are shown in Table 1. The first three datasets are datasets taken as is from related works focusing on hemolysis activity prediction with a wide range of modeling approaches applied to them, from feature-based approaches to deep learning-based approaches. This selection of diverse datasets allows AMPDeep’s performance to be compared to both the traditional methods of classification as well as the new sequence-based methods. This work also compiles a dataset from combining all three external datasets and properly splitting the train and test splits, to address the problem of similar sequences and bias in these datasets. The last dataset was gathered in this work using SwissProt [16], from peptides that have secretory keyword, as well as those within the cytoplasm that do not have this keyword for non-secretory peptides.

The amount of available training data for hemolytic activity prediction ranges from 800 data points to 2000 data points. While the amount of available training data for secretion prediction is nearly 30,000 data points. The relatively large amount of data that is available for secretory peptides is one of the reasons secretion prediction is chosen as the task to transfer from during transfer learning.

**Table 1:** Summary of all datasets used for training AMPDeep

Dataset	Origin	Train positive	Train negative	Test positive	Test negative
HLPpred-Fuse	Taken as is from [5]	433	423	663	1999
XGBC-Hem	Taken as is from [4]	442	442	110	110
RNN-Hem	Taken as is from [12]	1030	908	329	290
Combined	Compiled from [4, 5, 12]	901	901	50	50
Secretion	Aggregated from [16]	13950	14038	186	183

#### Keyword analysis results

**Fig. 2:**
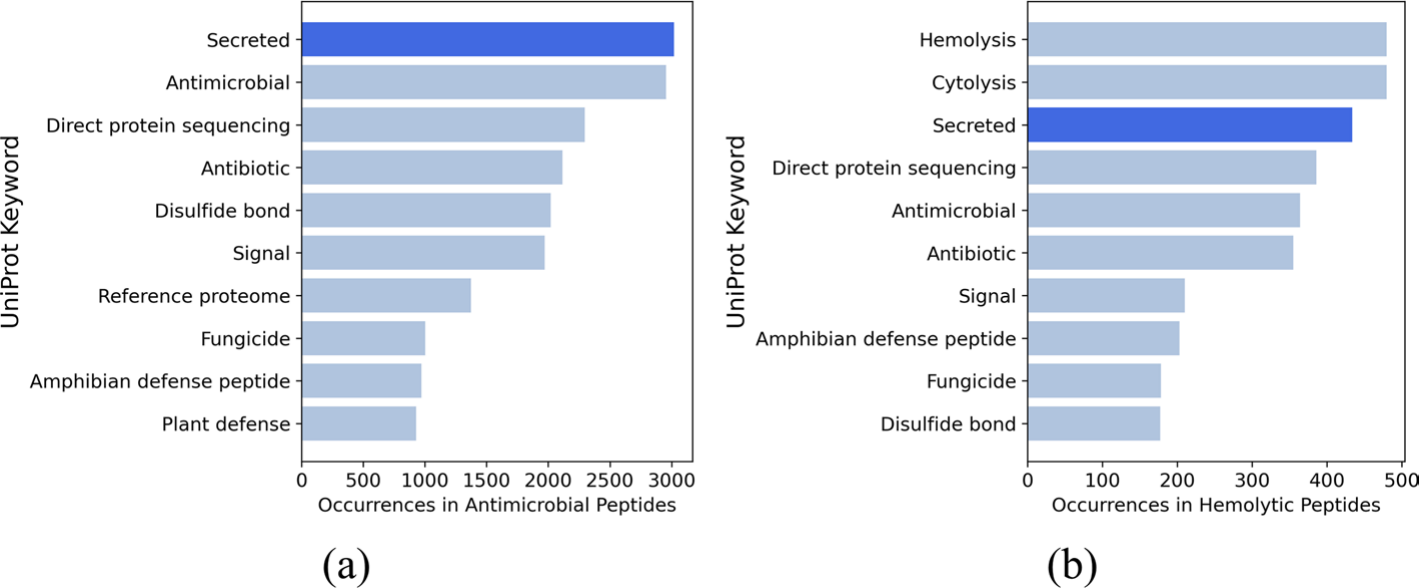
Top 10 keywords associated with: antimicrobial peptides (**a**), and hemolytic peptides (**b**) within the SwissProt Database

Analyzing the keywords acquired from UniProt’s reviewed peptides allows us to view the most prevalent properties of antimicrobial peptides as well as hemolytic peptides. To do so, all peptides within the SwissProt database that have are antimicrobial or hemolytic are acquired, creating two smaller datasets of antimicrobial peptides and hemolytic peptides respectively. The keywords associated with the peptides for each dataset are then counted, to find the most prevalent properties of these peptides. The code for extracting the keywords as well as analyzing them are included in the “keyword_analysis” script within the GitHub repository. The results are shown in Fig. 2. Further information regarding keywords of interest can be found in Additional File 1.

As it can be seen from Fig. 2, the most prevalent keyword associated with AMPs is “Secreted”. This co-occurrence also happens for hemolytic peptides, where a majority of the peptides are also secretory. This closeness in relationship was the main motivation for choosing secretory classification as a source task for transfer learning, justifying training on secretion detection data and then transferring the learned knowledge to the hemolysis activity prediction task.

#### The problem of sequence similarity

**Fig. 3:**
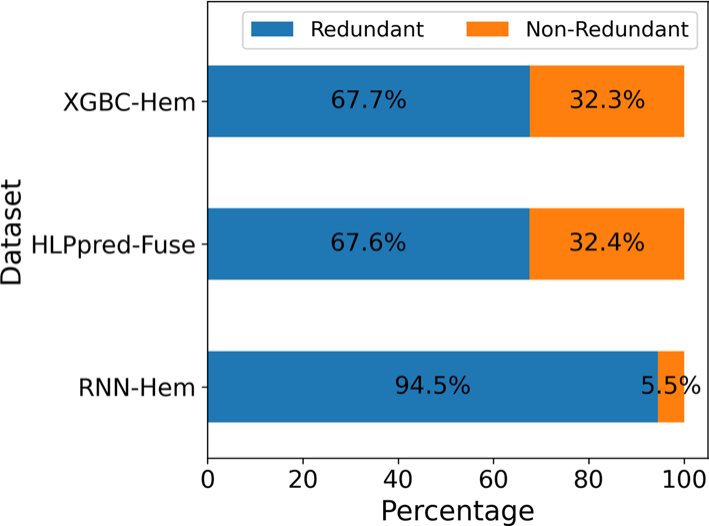
The composition of each dataset regarding representative and redundant sequences as calculated via CD-HIT with a threshold of 0.4

Analyzing the three acquired external datasets from [4, 5, 12] reveals that many similar sequences exist between their respective training sets and independent test sets. Existence of similar sequences in both train and test splits results in overestimating the performance of the model and creating impractical benchmarks. If a protein sequence has 40% or more similarity with another protein sequence with known function, then there is a high probability, that both proteins perform the same function [17]. The main source of this problem is the random splitting performed in these datasets to allocate sequences for the training and test splits. The degree of this redundancy in each dataset is shown in Fig. 3 calculated via CD-HIT.

As it can be seen from Fig. 3, all three external datasets suffer from a high degree of redundancy, which in turn with random splitting results in biased benchmarks for hemolytic activity prediction. To address this problem, this works compiles a dataset from combining all datasets, then using CD-HIT to remove the redundant sequences and removing any similar sequences that overlap between the test and train splits.

### Secretion prediction results

To start the training pipeline, transfer learning from a source model is needed. In this work, secretion detection is used as the source model. The model is first initialized on Prot-BERT-BFD [15], a protein language model trained on nearly 2 billion protein fragments, tasked with predicting amino acids that have been masked within the protein sequences. This model is then fine-tuned on secretory data using selective fine-tuning. As part of the hyper-parameter optimization, the pooling layer of the model is changed from the default BERT pooling to simple “Mean” pooling or “First Token” pooling. The results for these models are shown in Table 2.

**Table 2:** Results of training on the secretory dataset using different pooling mechanisms, validated on the secretory test set

Pooler	Hidden layer size	Accuracy	Recall	Precision	ROC-AUC	MCC
BERT Pooler	None	0.9431	0.9624	0.9275	0.9795	0.8868
Mean Pooling	32	0.9458	0.9731	0.9235	0.9827	0.8929
First Token Pooling	128	0.9566	0.957	0.957	0.9779	0.9133

As it can be seen from Table 2, the secretion prediction model is robust in regard to different hyper-parameters, with all pooling mechanisms resulting in similar performance. Having trained different secretory models, multiple transfer models are now available to be used as initializations for the next task of hemolytic activity prediction.

### Hemolysis prediction and transfer learning results

Different secretory models and architectures are used as initialization for training hemolysis prediction models using selective fine-tuning. Three datasets have been chosen to enable comparison of the results to previous works. For each dataset, the model is initialized on the secretion prediction model, then trained on 90% of the training dataset. Early stopping with patience of 10 is performed where the Mathews Correlation Coefficient (MCC) of the model on the remaining 10% of the training set is monitored, and the epoch with the highest MCC on this validation set is chosen as the best training epoch. The results are shown in Table 3 for XGBC-Hem dataset, in Table 4 for HLPpred-Fuse dataset, and in Table 5 for RNN-Hem dataset. All external results (non AMPDeep) in these comparisons are taken as reported in their respective works [4, 5, 12, 18, 19]. Following related works MCC is used as the main metric of evaluation.

**Table 3:** Hemolytic activity classification results for the model trained on XGBC-Hem [4] dataset’s train set and validated on its test set

Model	Accuracy	Recall	Precision	ROC-AUC	MCC
GBC [4]	0.904	–	0.865	0.905	0.809
LDA [4]	0.905	–	0.871	0.905	0.809
XGBC + Feature Selection [4]	0.923	–	0.885	0.923	0.846
AMPDeep	0.9863	0.9727	1	0.9995	**0.9731**

Bold values are for the best performing model

First three rows are reported as it was published in [4]

**Table 4:** Hemolytic activity classification results for the model trained on HLPpred-Fuse [5] dataset’s train set and validated on its test set. First three rows are reported as it was published in [5]

Model	Accuracy	Recall	Precision	ROC-AUC	MCC
HLPpred-Fuse [5]	–	0.845	–	0.967	0.823
HemoPI [5, 19]	–	0.804	–	0.952	0.754
HemoPred [5, 18]	–	0.652	–	–	0.34
AMPDeep	0.9369	0.8824	0.8667	0.9716	**0.8324**

Bold values are for the best performing model

**Table 5:** Hemolytic activity classification results for the model trained on RNN-Hem [12] dataset’s train set and validated on its test set

Model	Accuracy	Recall	Precision	ROC-AUC	MCC
SVM-Hem [12]	0.73	0.58	0.72	0.69	0.44
RF-Hem [12]	0.77	0.6	0.81	0.8	0.53
RNN-Hem [12]	0.76	0.76	0.7	0.87	0.52
AMPDeep	0.7997	0.8328	0.7988	0.8723	**0.5972**

Bold values are for the best performing model

First three rows are reported as it was published in [12]

As can be seen from Tables 3 and 4, using a large pre-trained transformer and training it on secretory and hemolytic sequences is superior to the state-of-the-art [4, 5], which extract physicochemical features from the input and classify it using traditional machine learning models. Table 5 further shows that AMPDeep can outperform a deep learning and sequence-based model, demonstrating the capabilities of this pipeline in learning from raw sequences. The hyper-parameters for all final models can be found in Additional File 1.

**Table 6:** Hemolytic activity classification results for the model trained on the combined dataset and validated on its independent test set

Model	Accuracy	Recall	Precision	ROC-AUC	MCC
AMPDeep	0.86	0.8	0.9091	0.8964	**0.7252**

Bold values are for the best performing model

Table 6 also report the performance of the model on the combined dataset. Unfortunately, this performance cannot be compared to related works, since this dataset was created from the combination of the external datasets and the test sequences overlap with external training sequences. However, the results on this dataset are reported, since to the best of authors’ knowledge this is the only hemolytic activity dataset where similar sequences between the train and test splits are removed.

### Selective fine-tuning results

Due to the small size of the training datasets (3K data points) compared to the large size of the model (400M parameters), restricting the number of parameters that are trainable can be beneficial to increase the stability of the training. To this end, this work introduces selective fine-tuning, where 8 different scenarios for freezing different parts of the model are considered for each experiment. The scenarios are shown in Table 7.

**Table 7:** Eight scenarios for selective fine-tuning

Scenario	Description
1	Full fine-tuning
2	Feature extraction
3	Pooler fine-tuning
4	Pooler replacement
5	Input embedding fine-tuning
6	Positional embedding fine-tuning
7	Input embedding + layer norm fine-tuning
8	Positional embedding + layer norm fine-tuning

**Fig. 4:**
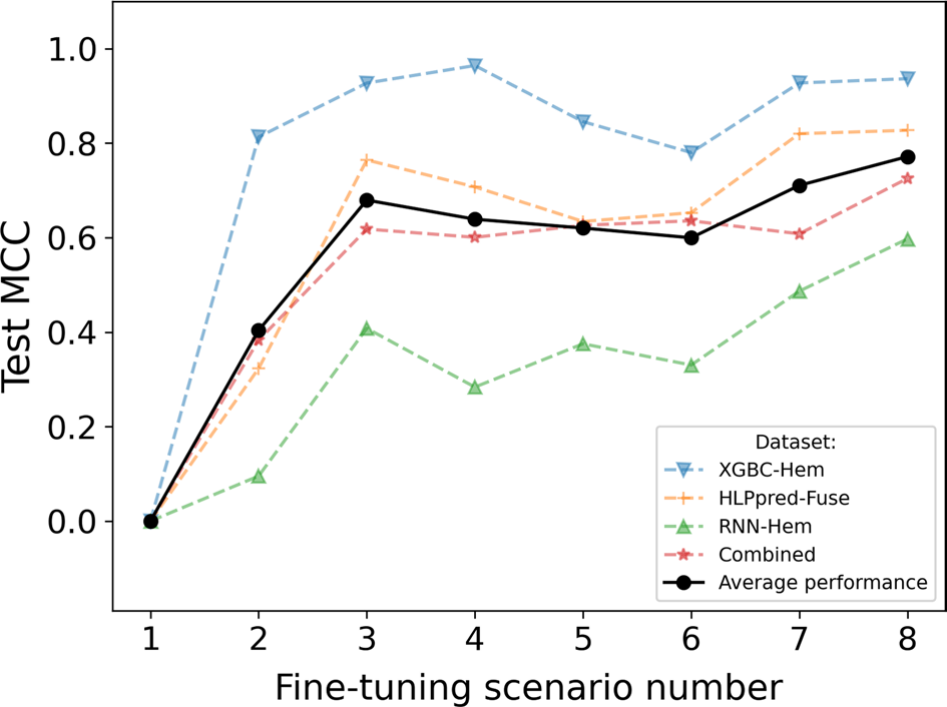
Model performance on 4 hemolytic datasets for 8 different scenarios of selective fine-tuning, and average performance on all 4. Scenario 8 has the highest performance on average for selective fine-tuning

Each of the scenarios defined in Table 7 was implemented for each of the four hemolytic datasets with hyper-parameter search for each model. The results for each dataset as well as an average of all results are demonstrated in Fig. 4.

**Fig. 5:**
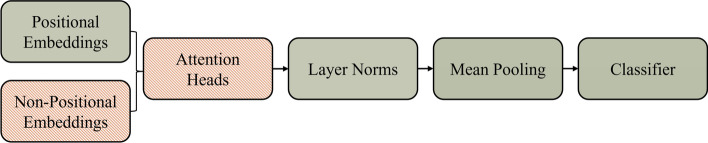
Overview of frozen (red and striped) and unfrozen (green and solid) parameters from selective fine-tuning for the final models

As it can be seen from 4, scenario 8 is the highest performing scenario on average. Therefore, the selective fine-tuning process determined that the best set of parameters to leave as unfrozen are the positional embedding parameters, the layer norm parameters, the pooler, and the classifier, while freezing the rest of the parameters i.e. the self-attention and the feed forward layers within the attention heads, and the non-positional embedding. These results are in line with findings from [20], where layer norm are left unfrozen during fine-tuning for natural language processing tasks. However, we found that freezing the non-positional embedding and swapping the pooling layer with mean pooling helps the final performance of the model. An overview of the frozen parameters are shown in Fig. 5.

### Ablation study–effect of transfer learning and selective fine-tuning

AMPDeep consists of multiple steps that assist in fine-tuning a large pre-trained model on a small number of peptide sequences. These steps include initialization on a pre-trained protein language model, modifying the pooling layer, selective fine-tuning, and transfer learning from secretion prediction. To assess the impact of each step on the final performance of the model, an ablation study is performed. During this study, the model initialization is compared between BERT [21] (a smaller transformer-based model pre-trained for masked token prediction on English text) and Prot-BERT-BFD [15]. Moreover, the pooling layer is compared between mean pooling and the default non-linear pooling that BERT offers. Furthermore, the fine-tuning procedure is compared between traditional fine-tuning and selective fine-tuning as proposed in this work. Finally, the effect of transfer learning from secretion prediction is studied. All models are trained on the XGBC-Hem benchmark with no hidden layer added before the classification layer. The results are shown in Table 8.

**Table 8:** Ablation results for different steps of the AMPDeep pipeline on the XGBC-Hem dataset

Approach	MCC
Baseline	0.846
BERT Init. (Natural Language) + Classification Layer Fine-Tuning	0.6639
BERT Init. (Natural Language) + Mean Pooling + Classification Layer Fine-Tuning	0.8091
Prot-BERT Init. + Mean Pooling + Classification Layer Fine-Tuning	0.8472
Prot-BERT Init. + Mean Pooling + Selective Fine-Tuning	0.9367
Prot-BERT + Mean Pool. + Secretion Transfer Learning + Selective Fine-Tun. (AMPDeep)	0.9731

As seen in Table 8 changing the pooling layer to be a simple mean pooling greatly increases the performance of the model. Moreover, changing the initialization to be from a pre-trained protein language model results in a performance similar to the baseline and adding selective fine-tuning, results in a large boost in this performance. Lastly, transfer learning from secretion prediction results in another large jump in MCC and the model achieving its final performance.

## Discussion

The main findings of AMPDeep are three-fold. Firstly, the techniques offered by AMPDeep for fine-tuning a protein language model on a small number of peptides are practical and result in superior empirical performance. These techniques include initialization from masked amino acid prediction, mean pooling, and selective-fine tuning. Secondly, bias was detected in three of the main datasets for hemolytic activity prediction and to that end, a new dataset was generated with proper data splitting regarding sequence similarity. Lastly, AMPDeep finds that transfer learning from secretion prediction of peptides is helpful to the task of hemolysis prediction. The similarities between the two data distributions of secretory peptides and hemolytic antimicrobial peptides, as seen in the keyword analysis section, might be one of the main reasons that transfer learning is effective. Overall, AMPDeep allows the knowledge learned from 2 billion protein fragments from Prot-BERT-BFD and learned from 30 thousand secretory peptides to be included in the training on hemolytic peptides. This knowledge is transferred in the form of initializations and arguably the patterns learned from masked amino acid prediction or secretory peptide prediction, are useful during training on hemolytic peptides and increase the stability of training, resulting in better performance. In the context of existing research, AMPDeep is the first deep learning-based model to out-perform the previous feature-based methods and this performance is owed mostly to the techniques applied during training and the transfer of pre-trained knowledge.

One of the possible limitations of this work is the use of keywords to find a secondary distribution to fine-tune the model on, i.e. secretory peptides. While this is intuitive, intuition does not necessitate positive transfer learning and the problem of negative transfer learning may occur if this method is applied to another task, e.g. peptide function prediction. This problem is still an active area of research and improvements can be done to move away from intuition and use empirical approaches to select initialization for transfer learning [22]. The second possible limitation of this study is its plethora of steps in order to fine-tune a large model. The number of sequential steps taken in AMPDeep to fine-tune the Prot-BERT-BFD model may be discouraging to others who wish to follow this work. We have included the scripts that were used for training within the Github repository and added simple control panels for them to facilitate the adoption of AMPDeep.

## Conclusions

In this work a hemolytic activity classification pipeline was implemented which leverages transfer learning from protein language modeling as well as secretory peptide prediction. Due to the prevalent secretory properties of AMPs, the model was first trained on secretory peptides then fine-tuned to predict hemolysis activity. The results show improved performance compared to the state-of-the-art on three different datasets ranging from feature-based models to deep learning-based models. Moreover, it is shown that unsupervised language modeling coupled with transfer learning and selective fine-tuning enable a large transformer model to be trained on a small amount of data and improve the performance on hemolysis prediction. Therefore, AMPDeep enables the adoption of large pre-trained protein language models for peptide classification, specifically hemolysis activity prediction, unlocking the use of many unlabeled data for supervised classification of a small number of peptide sequences. The methodology delineated in this work can enable fine-tuning large transformer models on small amount of amino acid-based sequence, which can impact other proteomic domains and facilitate adoption of large protein language models for these domains.

## Methods

### Data aggregation

In this work, three external hemolysis datasets are taken as is from the related literature to enable hemolysis prediction as well as comparison to the related works. These datasets are chosen from a variety of literature that include therapeutic peptides and antimicrobial peptides, and literature that use feature-based and deep learning-based methods for classification of hemolytic activity. This diversity in selection of datasets has been enforced to generate a fair comparison of AMPDeep’s performance to all types of existing approaches. Aside from the hemolysis data, one dataset is gathered for peptide secretion classification. This relatively larger dataset is used for training a source model for transfer learning. Overall, the categories of the datasets used in this work are as follows:

- *XGBC-Hem* dataset from [4], which in this work is referred to as XGBC-Hem, uses hemolysis prediction as part of a peptide generation pipeline. This work uses physicochemical properties to extract features from the data. Moreover, XGBC-Hem performs feature selection to improve the performance of the model. This dataset uses HemoPI-1 [19] which is created from experimentally validated peptides taken from the Hemolytik database [23], Swiss Prot (non-hemolytic peptides) [16], and Database of Antimicrobial Activity and Structure peptides (DBAASP v.2)[24]. The dataset was randomly divided into 80 and 20 percent for training and validation splits respectively.
- *HLPpred-Fuse* HLPpred-Fuse [5] is the current state-of-the-art model in hemolytic activity prediction and extracts biological features from the sequence and uses traditional machine learning models to classify the feature vectors. Similar to XGBC-Hem, this dataset uses HemoPI-1 as the training dataset. However, for evaluating the algorithm HLPpred-Fuse uses independent positive samples collected from the Hemolytik database and independent negative samples collected from PEPred-SUITE (randomly generated sequences) [25].
- *RNN-Hem* the dataset from [12], referred to in this work as RNN-Hem, is selected as the third dataset, since this work uses a sequence-based deep learning model to predict the hemolytic activity of peptides. This dataset uses the DBAASP database for annotations in regard to the activity of the peptide and their hemolytic properties. The data was further augmented with negative sequences collected from Swiss Prot, randomly generated sequences, and sequences created via shuffling the order of amino acids in positive sequences. For evaluation, RNN-Hem randomly splits the data into 75 and 25 percent splits for training and validation respectively.
- *Combined* the three datasets gathered for hemolytic activity prediction are combined to create a diverse dataset as well as to control the degree of sequence similarity between the training split and the test split, alleviating the problem of bias that exists in the external datasets.
- *Secretory data* the secretory peptides were gathered from UniProt’s reviewed proteins [16] with lengths of 200 and shorter, using the “Secreted” keyword. To acquire non-secretory peptides, the reviewed peptides were first narrowed based on their location to be in Cytoplasm, then secretory peptides were removed. The remaining peptides were randomly sampled to be nearly as many as secretory peptides. This data is split into 80%, 10%, and 10% splits for training, validation, and test sets. The test set is further refined using CD-HIT to remove all similar sequences to the training set and to create a fair test set for the secretory task. This problem of similarity is further studied in the following subsection.

#### Data preprocessing and sequence similarity detection

After each peptide sequence is acquired, the duplicate sequences are removed. Moreover, all of the peptides sequences that exist in both the hemolysis datasets and the secretory dataset, are removed from the secretory dataset to avoid leakage of hemolysis task into the transfer task. The peptide sequences are then edited to have one space between each amino acid, since this is the input scheme required by the model in later stages. The pre-processing used for all hemolytic benchmarks and the secretory data are included in the “preprocessing_hemolysis” and “preprocessing_secretion” scripts within the GitHub repository, respectively.

A high degree of similarity exists between the sequences in the training set and the test set of the hemolytic datasets. To assess this redundancy, first CD-HIT is used to find the representative sequences of each dataset and the redundant sequences. To alleviate this problem, in this work a combined dataset is compiled which does not share similar sequences between the train and the test splits. First, all hemolytic datasets are combined, and CD-HIT is used to remove all redundant sequences. Afterwards, from the remaining non-redundant sequences a test set is formed via taking 50 positive samples and 50 negative samples ( 10% of the data). The training set is then constructed from all sequences that are now similar to the sequences in the test set. The training set is then balanced via randomly removing negative data points. The process of removing similar data points is performed on the test set of the secretory data as well. Throughout this work CD-HIT was used with a threshold of 0.4 and number of words of 2.

### Model initialization

#### Language modeling and pre-training

The Prot-BERT-BFD [15] model is a transformer-based model with more than 400 million parameters, trained on 2 billion protein fragments. This model is inspired by BERT’s architecture [21], a popular transformer-based model in the natural language processing domain which uses the attention mechanism to create inner representations from an input sequence. BERT works through masked language modeling, which involves masking the a percentage of words in each sequence and predicting what the masked words should be. This pre-training strategy only requires sequences and does not require labels, and hence is self-supervised. Prot-BERT-BFD follows the same training strategy as BERT, however it does so on 2 Billion protein sequences and predicts masked amino acids. This unsupervised pre-training gives the model useful knowledge regarding protein sequences, which can in turn be useful for peptide classification. In this work, this large model with knowledge gained through pre-training on many protein sequences is adopted as initialization for the later stages. Prot-BERT-BFD was chosen as the initialization for two main reasons, firstly the size of the pre-training data, which is the largest available for all public models of such nature. Secondly, this model is easily available in the HuggingFace [26] repository and can be fine-tuned with ease. Aside from HuggingFace, the training scripts in this work utilize Python, Scikit-Learn, and PyTorch.

Prot-BERT-BFD is pre-trained as a language model, therefore masked language modeling heads are located at the last layer of the model. These heads are not suitable for outputting binary classification labels that are needed for hemolysis activity prediction, therefore the last layer of this model is removed and replaced with a fully connected neural network to act as the classifier.

#### Transfer learning from secretion prediction

Transfer learning has become a staple technique in deep learning model development, where one model is trained on a source dataset, then transferred to the target domain and fine-tuned on the target dataset. The source dataset is often larger than the target dataset, allowing the model to learn feature extraction before the transfer, then fine-tuning the learned feature space at the target domain after the transfer. Therefore, transfer learning can be viewed as an approach to pay the high data cost of deep learning’s automatic feature extraction at the source domain and stabilizing the training at the target domain.

In this work, transfer learning is applied from a model trained on peptide secretion prediction to a model prediction peptide hemolysis. The hope is that due to the similarity between secretory peptides distribution and hemolytic peptide distribution, transfer learning would transfer useful patterns learned from secretory peptides to the hemolysis classification model. Aside from the data distribution similarity which we study in the form of keyword analysis, the data size for the available secretory peptides are much larger than hemolytic peptides, making secretory peptide prediction a suitable source domain for transfer learning.

### Model training

#### Hyper-parameter optimization

**Fig. 6:**
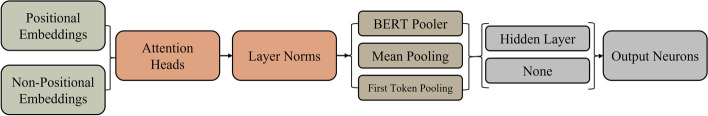
Overview of model architecture and its variations. The pooling layer can be swapped between three possible pooling mechanism. The classification layer can also be modified to insert a hidden layer

To optimize the performance of the model and try different architectures, two layers are modified within the model. Firstly, the pooling layer is toggled between default BERT-based pooling layer, mean-pooling layer, and first token pooling layer. Secondly, the fully connected network at the end of the model tasked with classification is assessed in term of the size of the hidden layer and its number of neurons. To do so, after the model is chosen and initialized, a fully connected layer is attached to the last layer of the model to prepare it for sequence classification. The size of the fully connected layer, the type of the pooling layer, as well as the learning rate for training are determined using a grid search hyper-parameter optimization. This optimization can be performed through the control panel provided in the script “training” in the GitHub repository. The model architecture and the possible variations of it are shown in Fig. 6.

To determine the optimum length of training, early-stopping with patience of 10 epochs is used. To create a fair monitoring metric, 10% of the training set for each benchmark is isolated as the validation set. The model is first trained on the secretory task, with early stopping monitoring the secretory validation set’s MCC. After the model is trained, the weights are transferred to a new model for training on hemolysis classification task. As the weights are transferred, the model is trained similar to last stage, with early stopping monitoring the validation set performance. The range of hyper-parameters explored during hyper-parameter optimization can be found in Additional File 1.

#### Selective fine-tuning and parameter freezing

Since Prot-BERT-BFD is a rather large model with more than 400 million parameters, fine-tuning it with datasets as small as 800 data points proved to be challenging. As seen in previous related deep learning-based works [12, 14], when a small number of hemolytic peptides are used for training a large deep learning-based model, the automatic feature extraction may fail to fully learn useful representation that can outperform biological or physicochemical features. In this work, to alleviate this problem two techniques are implemented; transferring learned features, and making the effective number of parameters in the model smaller. Firstly transfer learning is used to initialize the model with favorable weights and features. Secondly, a novel approach for fine-tuning the model is introduced, called selective fine-tuning, where many parameters of the model are frozen during training and are not changed via the stochastic gradient descent. Through freezing the parameters, the number of trainable parameters fall, resulting in less number of data points needed during training.

AMPDeep first sections the parameters of the model into the following categories: positional embedding, non-positional embedding, attention heads, layer norms, pooler, and classifier parameters. Each category of the model can either be frozen during training, or left unfrozen to be changed. This decision (to freeze or not) is treated as a hyper-parameter and is toggled for each category of parameters during hyper-parameter optimization, allowing “selective fine-tuning” to take place. To this end, 8 scenarios are defined and investigated as selective fine-tuning:

- *Scenario 1* Full fine-tuning: the standard method of fine-tuning where all parameters of the model are unfrozen and trainable.
- *Scenario 2* Feature extraction: the typical method of fine-tuning when small amount of data is available, where only the classification layers are trainable.
- *Scenario 3* Pooler fine-tuning: Pooler plays an important role passing on the extracted features to the classifier layer. In this scenario pooler is also fine-tuned.
- *Scenario 4* Pooler replacement: In our experiments through trial and error the pooling layer was found to be highly impactful on the performance of the model. Therefore, in this work the effect of pooling layer is investigated via swapping the layer with simple mean or first token pooling mechanisms. From this scenario onward, pooler is always replaced with either mean or first token pooling.
- *Scenario 5* Input embedding fine-tuning: The input embedding is how the sequence is embedded in terms of its context and its order to be passed on to the model. In this scenario, all of the embedding vectors are unfrozen and can be fine-tune, allowing the model to adapt to the different inputs domain of the data. Mean pooler is used and classification layer is also unfrozen.
- *Scenario 6* Positional embedding fine-tuning: This embedding is in charge of representing the order of the sequence. In this scenario it is left unfrozen. Mean pooler is used and classification layer is also unfrozen.
- *Scenario 7* Input embedding + layer norm fine-tuning: In this scenario all of the embedding vectors as well as the layer norms are fine-tuned. Mean pooler is used and classification layer is also unfrozen.
- *Scenario 8* Positional embedding + layer norm fine-tuning: In this scenario the positional embedding vector as well as the layer norms are fine-tuned. Mean pooler is used and classification layer is also unfrozen.

This type of fine-tuning enables a greater control over the parameters of the model when compared to traditional fine-tuning. This process is inspired by the fine-tuning process described in [20], where positional embedding layer and the layer norms are unfrozen, while the rest of the parameters are frozen. We further add to this strategy via exploring more freezing scenarios and adding more control over the type of the pooler. The different scenarios and their parameters are available on GitHub through the script “plot_results”.

### Evaluation metric

Following the related works, in this work the Mathews Correlation Coefficient is used for evaluating the performance of the models. This metric is a used to measure the association between the binary vector of labels and the binary vector of predictions and is defined as shown in Eq. (1) where *TP*, *TN*, *FP*, and *FN* denote True Positive, True Negative, False Positive, and False Negative respectively. Higher MCC demonstrates higher degree of agreement between the ground truth and the predicted labels, therefore, the model with highest MCC on the validation set is chosen for inference on the test set and final evaluation.

1 $$MCC = \frac{{TP \times TN - FP \times FN}}{{\sqrt {\left( {TP + FP} \right)\left( {TP + FN} \right)\left( {TN + FP} \right)\left( {TN + FN} \right)} }}$$

## Supplementary Information

**Additional file 1.** A Word document containing the additional methodology and results.

## Data Availability

The data and the scripts for this work are available through GitHub at https://github.com/milad73s/AMPDeep, and archived at Zenodo at https://zenodo.org/record/6992526.
