# Supplementary material for: AMPDeep: hemolytic activity prediction of antimicrobial peptides using transfer learning
Source: BMC Bioinformatics. 2022 Sep 26;23:389. doi: 10.1186/s12859-022-04952-z (PMC9511757; doi:10.1186/s12859-022-04952-z)
Supplement: Supplementary file 1 — Additional file 1. A Word document containing the additional methodology and results. [file 12859_2022_4952_MOESM1_ESM.docx]

**Supplementary Materials – AMPDeep**

**Keyword Analysis – Antimicrobial Peptides**

To find the peptides within the SwissProt database that are antimicrobial, first all sequences with length of 200 or less amino acids are identified. The sequences that had the following keywords where denoted as antimicrobial and were chosen for keyword analysis through keyword association and count:

'Antimicrobial', 'Antibiotic', 'Antiviral defense', 'Antiviral protein', 'Fungicide', 'Tumor suppressor', 'Antiviral protein', 'Plant defense', 'Amphibian defense peptide', 'Defensin', 'Bacteriocin', 'Lantibiotic', 'Innate Immunity'

**Hyper-Parameter Optimization**

To find the best set of parameters for training, hyper-parameters were changed to cover a wide range of values as shown in Table S1.

Table S1 – Hyper-parameter value range during hyper-parameter optimization.

| Parameter | Possible Values | Parameter | Possible Values |
| --- | --- | --- | --- |
| Initialization | BERT or Prot-BERT-BFD | Classifier Hidden Layer Size | None, 32, 128, 1024 |
| Number of Epochs | 50 (+ early stopping) | Pooling Mechanism | BERT pooling, mean pooling, first token pooling |
| Initial Learning Rate | 0.0005, 0.00005 | Batch Size | 32 |
| Positional Embeddings | Frozen, unfrozen | Non-Positional Embeddings | Frozen, unfrozen |
| Attention Heads | Frozen, unfrozen | Layer Norm | Frozen, unfrozen |
| Pooler | Frozen, unfrozen | Classifier | Unfrozen |

**Best Set of Hyper-Parameters**

After the hyper-parameters are optimized for each benchmark, the final hyper-parameters are shown in Table S2. Selective fine-tuning denotes freezing all parameters except positional embedding, layer norms, pooler, and classification layer.

Table S2 – Final hyper-parameters for each benchmark.

| Benchmark | Pooling Mechanism | Classifier Hidden Layer Size | Initial Learning Rate | Selective Fine-Tuning |
| --- | --- | --- | --- | --- |
| XGBC-Hem | Mean | None | 0.0005 | Yes |
| HLPpredFuse | Mean | 1024 | 0.0005 | Yes |
| RNN-Hem | Mean | None | 0.0005 | Yes |
| Combined | Mean | 128 | 0.0005 | Yes |
